# LncRNA NEAT1 is upregulated in recurrent aphthous stomatitis (RAS) and has predictive values

**DOI:** 10.1186/s12903-021-01909-1

**Published:** 2021-12-31

**Authors:** Yaolun Han, Lu Wang, Qingfu Li, Hongli Chen, Xin Ma

**Affiliations:** Department of Stomatology, Henan Provincial People’s Hospital, No. 7 Weiwu RoadHenan Province, Zhengzhou City, 450000 People’s Republic of China

**Keywords:** Recurrent aphthous stomatitis, NEAT1, IL-2, IL-1β, TNF-α

## Abstract

**Background:**

LncRNA NEAT1 promotes inflammatory responses, which contribute to recurrent aphthous stomatitis (RAS). This study focused on the involvement of NEAT1 in RAS.

**Methods:**

RT-qPCR and ELISA were performed to determine the expression of NEAT1 and proinflammatory factors (IL-2, IL-1β, and TNF-α) in plasma from patients with a history of RAS and showing symptom (n = 80, S-RAS group), people with a history of RAS but showing no symptom (n = 80, NS-RAS group), and controls without a history of RAS (n = 80, Control group). Correlation analysis was performed with Pearson’s correlation coefficient. S-RAS group received treatmen,t and plasma levels of NEAT1 and proinflammatory factors were compared before and after treatment. S-RAS group was followed up for 12 months, and the recurrence was recorded.

**Results:**

Plasma NEAT1, IL-2, IL-1β, and TNF-α levels were the highest in the S-RAS group, followed in turn by NS-RAS and control groups. NEAT1 was positively and significantly correlated with IL-2, IL-1β, and TNF-α across S-RAS and NS-RAS samples, but not control samples. After treatment, plasma levels of NEAT1, IL-2, IL-1β, and TNF-α decreased significantly. Moreover, a higher recurrence rate was observed during the follow-up in patients with high plasma NEAT1 levels.

**Conclusion:**

NEAT1 is upregulated in RAS and correlated with multiple proinflammatory factors. Moreover, NEAT1 has predictive values for RAS.

## Background

Recurrent aphthous stomatitis (RAS), presenting as shallow, painful round ulcer with yellowish-gray center and erythematous margin (well-defined), is the most frequently diagnosed oral mucosa ulcerative disease [1, 2]. The incidence of RAS varies a lot across different populations. Globally, about 2–66% of the population are suffering from RAS [3]. RAS is closely correlated with brushing habits, brushing time, other oral diseases, age, and exercise time, while its pathogenesis remains unclear [4]. Multiple drugs have been used to control symptoms of RAS, including colchicine, clofazimine, and pentoxifylline [5–7]. However, the recurrence rate is high. It is estimated that more than 50% of RAS will experience recurrence with 3 months after treatment [5]. Therefore, novel preventative and treatment approaches are still needed.

RAS is an inflammatory condition in oral mucosa with the involvement of multiple inflammatory factors, such as IL-2, IL-1β, and TNF-α [8–10]. Therefore, controlling the production of inflammatory responses is critical in the treatment of RAS. Long non-coding RNAs (lncRNAs), which lack protein-coding capacity, affect the downstream expression of other non-coding RNAs and protein synthesis to participate in human diseases [11]. Especially, lncRNAs may interact with multiple inflammation pathways to regulate inflammatory responses in the human body [12, 13]. Therefore, lncRNAs may serve as potential diagnostic markers and therapeutic targets for RAS. However, the involvement of lncRNAs in RAS remains hardly known. LncRNA NEAT1 is a critical player in cancer biology [14, 15]. It is involved in the development and progression of multiple cancers, such as colorectal cancer and breast cancer. NEAT1 regulates cell behaviors, such as proliferation, apoptosis, and invasion to accelerate cancer growth and metastasis. Besides cancers, NEAT1 also participates in other physiological and pathological processes, such as inflammatory response [16], which contributes to RAS. Therefore, in this study, we explored its involvement in RAS.

## Methods

### Participants and plasma samples

To study the differential expression of NEAT1, IL-2, IL-1β, and TNF-α in RAS, plasma samples were donated by patients with a history of RAS and showing symptom (n = 80, S-RAS group, 40 females and 40 males, 44.4 ± 6.1 years), people with a history of RAS but showing no symptom (n = 80, NS-RAS group, 40 females and 40 males, 44.6 ± 6.0 years), and controls without a history of RAS (n = 80, Control group, 40 females and 40 males, 44.3 ± 5.8 years). These three groups showed similar distributions of age and gender. Changes in pH, TNF-α level, and salivary nitric oxide contents were observed in S-RAS patients, but not in NS-RAS patients were compared to the controls. All participants were enrolled at Henan Provincial People's Hospital from May 2019 to October 2020. This study excluded patients with other severe inflammatory diseases, diabetes, malignancies, heart diseases, and so on. This study did not include pregnant women and patients who were taking medications. All participants signed informed consent. This study was approved by the Ethics Committee of Henan Provincial People's Hospital (Approval No. H3#653) and conducted following the guideline of the Declaration of Helsinki.

### Therapies, follow-up, and plasma preparations

All patients in the S-RAS group received 1.5 mg/day colchicine treatment. No obvious symptoms were observed after 3 weeks of treatment. During the treatment, taking medications or any beverages containing alcohol was not allowed. Blood samples (about 5 ml) were extracted from each participant in the S-RAS group, NS-RAS group, and control group prior to the treatment on the day of admission. Blood samples were also extracted from each patient in the S-RAS group after treatment. After treatment, all 80 participants in the S-RAS group were followed every month for a total of 12 months. Blood was also extracted during the follow-up on the day of diagnosis. Blood samples were transferred to EDTA tubes and centrifuged for 10 min at 1200 g to obtain plasma samples. About 3 ml of plasma was isolated from each blood sample. All plasma samples were kept in liquid nitrogen prior to the subsequent assays.

### ELISA

Human IL-2 Quantikine ELISA Kit (D2050, R&D Systems), human IL-1 beta/IL-1F2 Quantikine ELISA Kit (DLB50, R&D Systems), and human TNF-alpha DuoSet ELISA kit (DY210-05, R&D Systems) were used to determine the levels of IL-2, IL-1β, and TNF-α in plasma samples from each participant, respectively. All operations were performed following the manufacturer’s instructions.

### RNA sample preparations

Total RNAs were extracted from each participant using Direct-zol reagent (ZYMO RESEARCH) and treated with DNase I for a total of 120 min to remove DNA contaminations. RNA concentration and integrity were analyzed using a 2100 Bioanalyzer. RNA samples with RIN values higher than 8.5 were achieved in each sample. Otherwise, RNA isolation and digestion were repeated.

### RT-qPCR

With 3000 ng total RNA as template, cDNA synthesis was performed through reverse transcriptions (RTs). QPCRs were then performed with cDNA samples to determine NEAT1 expression level with 18S rRNA as the internal control. All Ct values were normalized using the 2^−ΔΔCt^ method.

### Statistical analysis

Data analysis and image preparation were performed using GraphPad Prism 6 software (GraphPad, USA). The paired test was performed to compare pre- and post-treatment data. Correlations were performed using Pearson’s correlation coefficient. Three independent groups were compared by ANOVA Tukey’s test. The 80 participants in the S-RAS group were divided into high and low NEAT1 level group median post-treatment plasma NEAT1 level as the cutoff (n = 40 per group). RAS-free curves were plotted using the follow-up data and compared by log-rank test. *P* < 0.05 was a significant difference.

## Results

### Analysis of plasma levels of NEAT1, IL-2, IL-1β, and TNF-α in three groups of participants

**Fig. 1:**
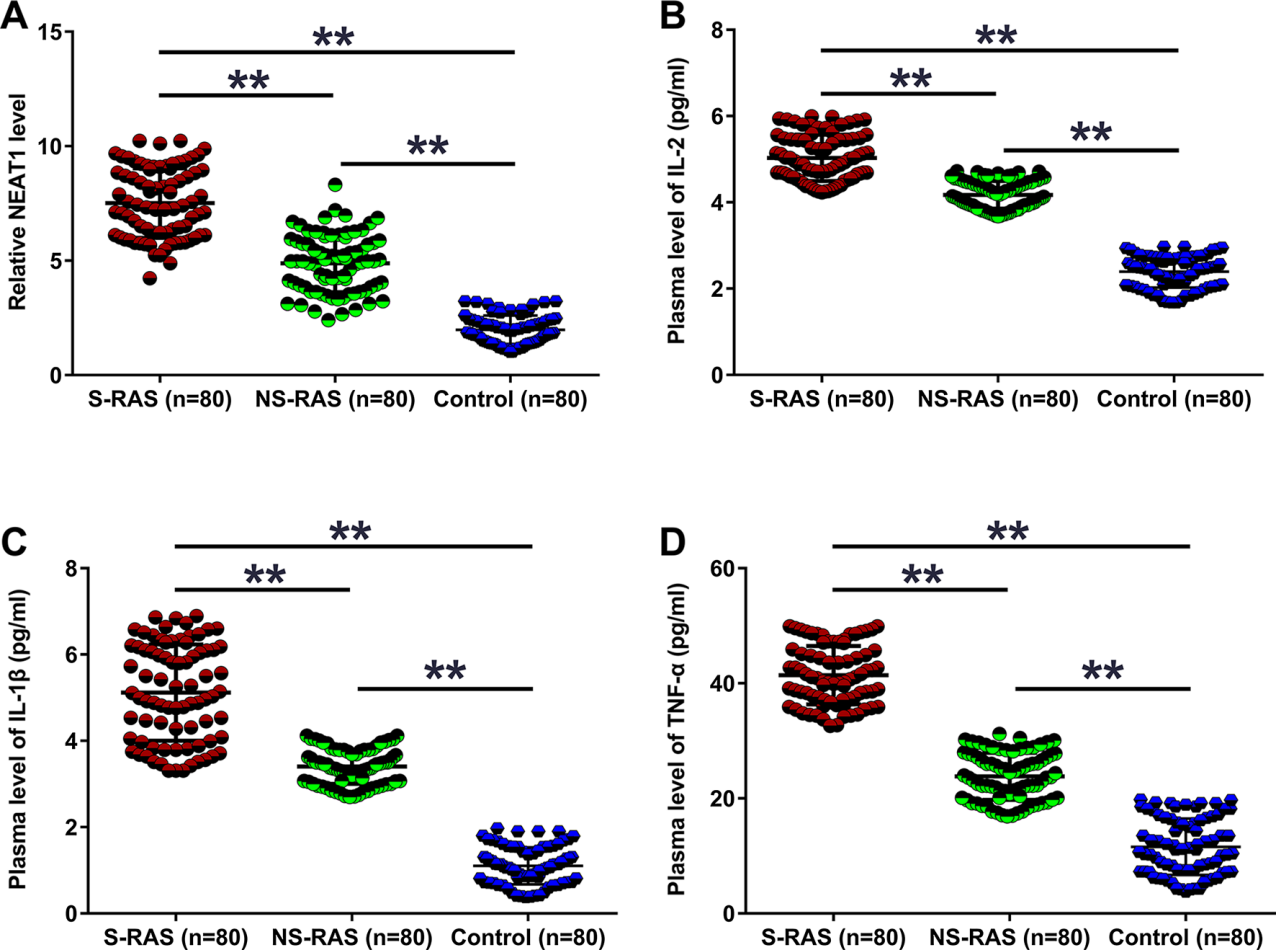
Analysis of plasma levels of NEAT1, IL-2, IL-1β, and TNF-α in three groups of participants. Plasma samples from the S-RAS, NS-RAS, and control groups were subjected to RT-qPCR and ELISA to determine the plasma levels of NEAT1 (**a**), IL-2 (**b)**, IL-1β (**c**), and TNF-α (**d**). **p < 0.01

Plasma samples from the S-RAS, NS-RAS and control groups were subjected to RT-qPCR and ELISA to determine the plasma levels of NEAT1, IL-2, IL-1β, and TNF-α. Our results illustrated that plasma levels of NEAT1 (Fig. 1a, *p* < 0.01), IL-2 (Fig. 1b, *p* < 0.01), IL-1β (Fig. 1c, *p* < 0.01), and TNF-α (Fig. 1d, *p* < 0.01) were the highest in the S-RAS group, followed in turn by the NS-RAS and control groups. Therefore, increased plasma NEAT1, IL-2, IL-1β, and TNF-α levels may participate in RAS.

### Correlations of NEAT1 with IL-2, IL-1β, and TNF-α

**Fig. 2:**
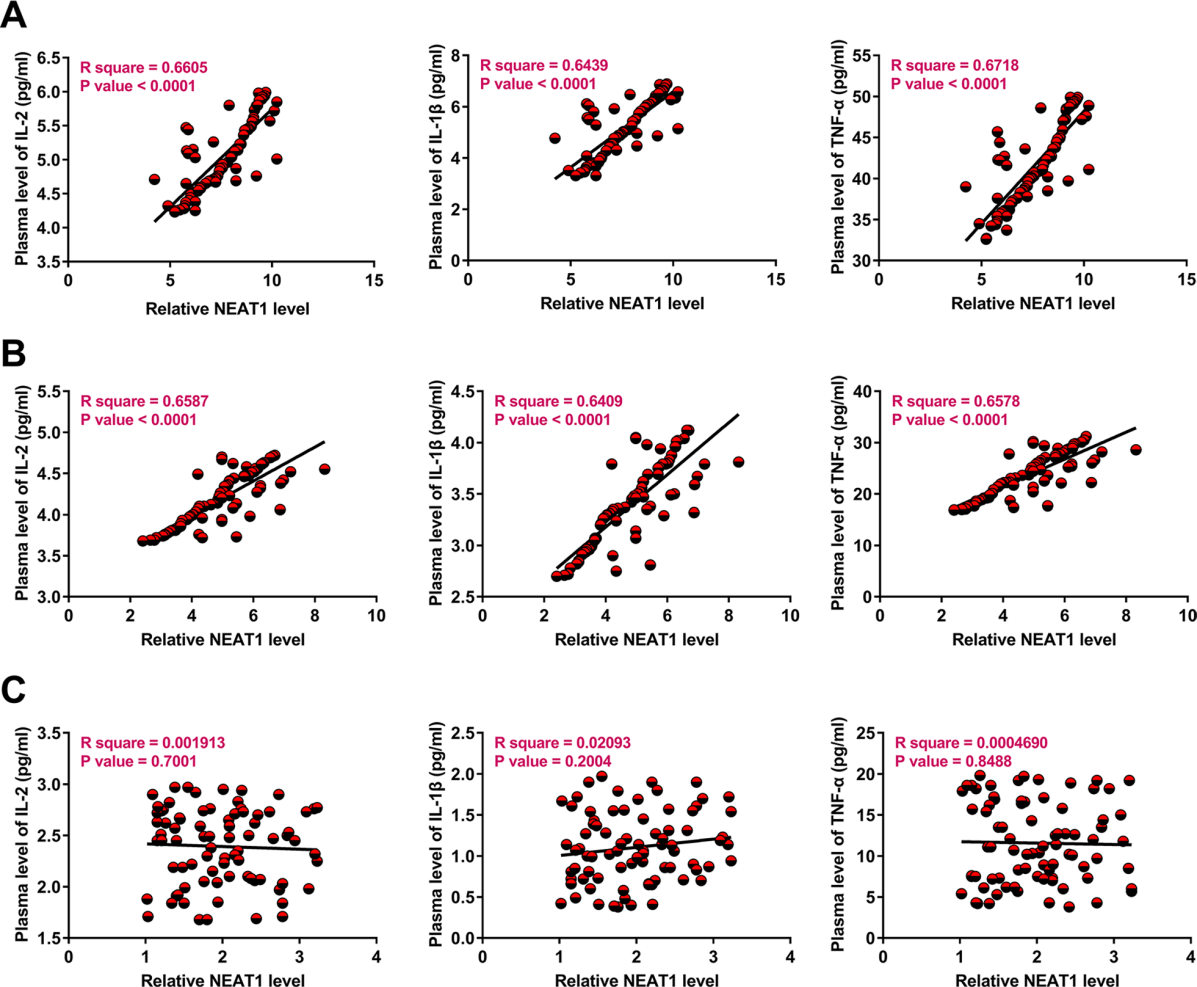
Correlations of NEAT1 with IL-2, IL-1β, and TNF-α. Correlations of NEAT1 with IL-2, IL-1β, and TNF-α across the S-RAS (**a**), NS-RAS (**b**), and control (**c**) samples were analyzed using Pearson’s correlation coefficient

Correlations of NEAT1 with IL-2, IL-1β, and TNF-α across S-RAS (Fig. 2a), NS-RAS (Fig. 2b), and control (Fig. 2c) samples were analyzed using Pearson’s correlation coefficient. It was observed that NEAT1 was positively and significantly correlated with IL-2, IL-1β, and TNF-α across the S-RAS and NS-RAS samples, but not the control samples. Therefore, NEAT1 may be involved in inflammatory responses in RAS.

### Comparison of plasma NEAT1, IL-2, IL-1β, and TNF-α levels before and after treatment

**Fig. 3:**
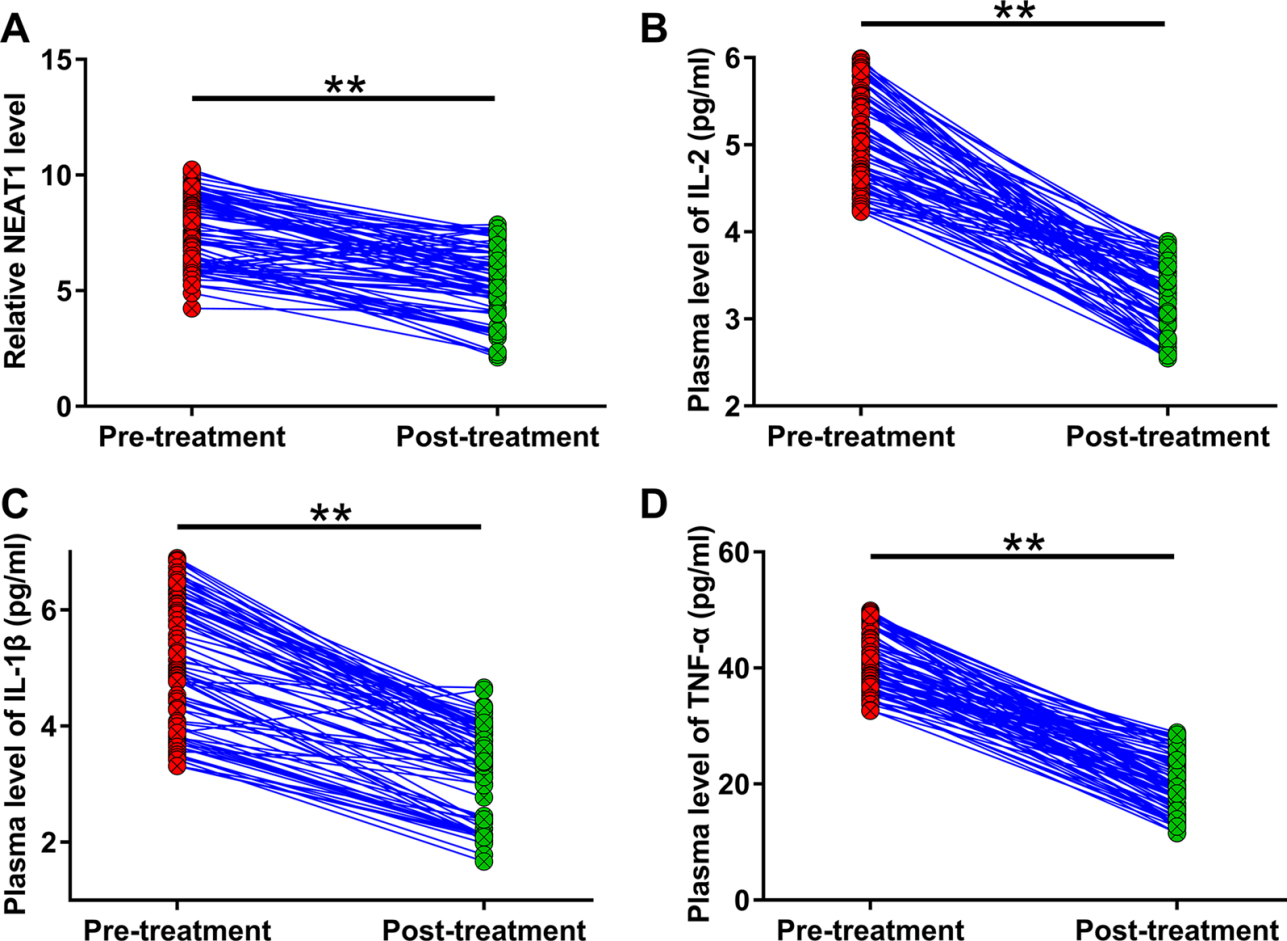
Comparison of plasma levels of NEAT1, IL-2, IL-1β, and TNF-α before and after treatment. Plasma levels of NEAT1 (**a**), IL-2 (**b**), IL-1β (**c**), and TNF-α (**d**) were measured before (pre-treatment) and after (post-treatment) treatment. Paired t test was applied to compare pre- and post-treatment data. **p < 0.01

In this study, plasma NEAT1, IL-2, IL-1β, and TNF-α levels were measured before (pre-treatment) and after (post-treatment) treatment. It was observed that plasma NEAT1 (Fig. 3a), IL-2 (Fig. 3b), IL-1β (Fig. 3c), and TNF-α (Fig. 3d) levels decreased significantly after treatment (*p* < 0.01). Therefore, plasma NEAT1, IL-2, IL-1β, and TNF-α levels may serve as indicators of the treatment of RAS.

### Analysis of the predictive value of plasma NEAT1 in RAS

**Fig. 4:**
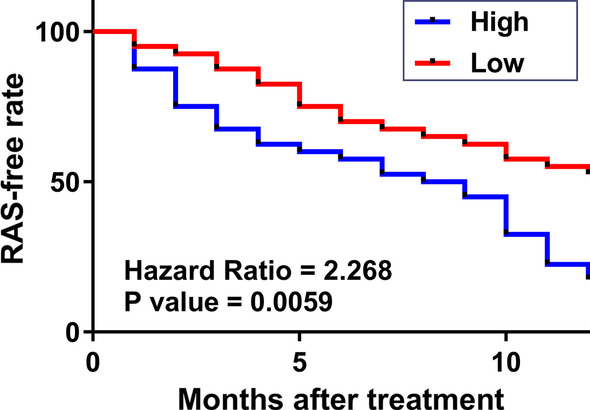
Analysis of the predictive value of plasma NEAT1 in RAS. All 80 participants in the S-RAS group were divided into high and low NEAT1 level groups (n = 40, cutoff value = median post-treatment plasma level of NEAT1). RAS-free curves were plotted using the follow-up data and compared using the log-rank test

The 80 participants in the S-RAS group were divided into high and low NEAT1 level groups (n = 40, cutoff value = median post-treatment plasma NEAT1 level). RAS-free curves were plotted using the follow-up data and compared using the log-rank test. A higher recurrence rate was observed during the follow-up in patients with high plasma NEAT1 levels (Fig. 4).

## Discussion

In this study, we explored the involvement of NEAT1 in RAS and analyzed its diagnostic value. We showed that NEAT1 was highly expressed in RAS and correlated with multiple inflammatory factors. In addition, plasma NEAT1 may serve as a predictive factor for RAS.

Besides the oncogenic role of NEAT1 in cancer biology [17], it also participates in multiple inflammatory diseases [16, 18]. It was reported that NEAT1 interacts with the axis of Let-7a/TLR4 to promote inflammatory responses, thereby accelerating the progression of liver injury involved in sepsis [16]. NEAT1 can also activate inflammasomes in macrophages, promoting the development of peritonitis and pneumonia [18]. RAS is also an inflammatory process, while the involvement of NEAT1 in RAS is unknown. In this study, we observed NEAT1 upregulation in RAS. After treatment, NEAT1 was significantly downregulated. Moreover, RAS patients in the high NEAT1 group experienced a significantly higher incidence rate of RAS during the follow-up. Therefore, NEAT1 is involved in RAS, and the changes in plasma NEAT1 may be used as a maker to monitor the treatment outcomes of RAS and predict the recurrence of RAS. RAS patients with high NEAT1 levels after treatment should be particularly treated to avoidrecurrence. However, patients in the present study were treated with colchicine, which has been questioned by previous studies and also interferes with inflammasomes [19, 20]. Therefore, whether the changes in NEAT1 expression are caused by the recovery of RAS or the application of colchicine remains to be further explored.

Interestingly, our study also observed the upregulation of NEAT1 and multiple inflammatory factors, including IL-2, IL-1β, and TNF-α, in the NS-RAS group, which included RAS patients without apparent symptoms. Therefore, people with a history of RAS may experience continuously inflammatory responses. Therefore, controlling inflammatory responses may be applied to prevent the occurrence of RAS.

In this study, we showed that NEAT1 was closely correlated with IL-2, IL-1β, and TNF-α across the S-RAS and NS-RAS samples, but not the control samples. Therefore, NEAT1 may regulate the production of IL-2, IL-1β, and TNF-α to promote the occurrence of RAS. This study was performed to analyze the involvement of NEAT1 in RAS. We found that NEAT1 may be used as a molecular marker to predict the treatment outcomes and the recurrence of RAS. However, the reliability of the prediction remains to be further tested by more clinical studies with larger sample sizes. Moreover, this study observed significant correlations of NEAT1 with IL-6, IL-18, and TNF-α only in the S-RAS and NS-RAS patients but not in the control participants. Therefore, we speculate that some pathological mediators may mediate the interaction of NEAT1 with IL-6, IL-18, and TNF-α. However, the mechanisms underlying their potential interactions are still unclear. Future studies are needed to explore the possible mechanisms and the role of immune status and stress levels in RAS.

## Conclusion

NEAT1 was highly expressed in RAS, and it predicts the treatment outcomes and recurrence of RAS after treatment. Moreover, NEAT1 may promote the production of multiple inflammatory factors to induce RAS.

## Data Availability

The datasets generated during and/or analyzed during the current study are available in https://www.jianguoyun.com/p/DaJm0gcQ58LcCRi4goUE.
